# Mental Health in Australia and the Challenge of Community Mental Health Reform

**DOI:** 10.17816/CP44

**Published:** 2021-03-20

**Authors:** Sebastian Rosenberg, Carol Harvey

**Affiliations:** The Australian National University; University of Sydney; University of Melbourne

**Keywords:** Community mental health, psychosocial community support, clinical mental health, national mental health policy

## Abstract

Australia was one of the first countries to develop and implement a national mental health plan, 30 years ago. This national approach belied the countrys federal structure, in which the federal government takes responsibility for primary care while state and territory governments manage acute and hospital mental health care. This arrangement has led to significant variations across jurisdictions. It has also left secondary care, often provided in the community, outside of this governance arrangement. This article explores this dilemma and its implications for community mental health, and suggests key steps towards more effective reform of this vital element of mental health care.

## INTRODUCTION

Australia can point to repeated evidence ranking its health care system as one of the most effective in the world [1]. However, such assessments typically do not take mental health care into account. More recent analysis of international comparative data suggests the performance of Australia’s mental health system is mixed at best [2]. One of the key reasons for this mixed performance has been a limited commitment to community mental health care.

As this article will demonstrate, despite early promising beginnings, Australia’s approach to mental health care has become increasingly fragmented and chaotic. While responsibility between governments is clear in relation to primary and tertiary levels of care, secondary care, typically provided in the community, has languished. As a result, for people with mental health problems deemed too complex for primary care, there is often little choice but to go to hospital and they may not receive community mental health services unless they are either acutely or severely unwell. A recent Victorian Auditor General report confirmed that area public mental health services only see ‘the most unwell’ people, creating significant service problems in other parts of the mental health ‘system’ [3]. A national approach to hospital avoidance and early intervention in the community has failed to emerge. This has resulted in large service gaps.

This article will review how this situation has developed. It will first provide an overview of the complicated arrangements by which Australia’s nine governments share responsibility for different aspects of mental health care. The article will then give an overview of developments in community mental health care, particularly in the early stages of national commitment to mental health policies and plans. We will then provide an explanation of the current problems affecting community mental health care and point to some of the key issues to be resolved if progress is to be resumed. There is little doubt that the development of a robust and well-organized system of community mental health care is central to future national mental health reform efforts.

It is not possible to understand the Australian context without some appreciation of its political system. There are eight state or territory governments and one federal (national) government. Responsibility for health care, including mental health care, is split between state/territory and federal governments. The federal government is responsible for the national system of public health insurance, the Medicare Benefits Scheme (MBS) and the Pharmaceutical Benefits Scheme (PBS), which subsidizes medications. The MBS covers primary and allied health care in the community, particularly those services provided by general practitioners. The states and territories manage hospital-based health care, including emergency, inpatient and outpatient services. Australia’s constitution provides the states and territories with autonomy in relation to health care, including mental health [4]. This has given rise to some variation between jurisdictions, for example, the mental health system of New South Wales (NSW) looks different to that of Victoria. Part of this difference is about how jurisdictions respond to their geography and demography, but it also reflects policy, funding and service choices made over time. Despite these regional differences, it is possible to see some important national trends in relation to community mental health. These will be the focus of this article.

## MENTAL HEALTH CARE IN AUSTRALIA

Australian community-based mental health care developed gradually in the twentieth century, especially after World War Two [5]. This period saw the uncoordinated development of community clinics as well as community psychosocial support services, emerging from the charitable and welfare sectors. Australia was one of the first countries to embark on a national mental health strategy, with the first National Mental Health Policy published in 1992 [6]. This progressive document referred to several key principles, including the rights and civil liberties of consumers and carers. A key goal was to enable the states and territories to close the long-term psychiatric institutions, thus permitting people with persistent mental illness to live in the community.

For this to occur, it would be necessary to close the old asylums and replace them ‘with a mix of general hospital, residential, community treatment and community support services’ [6]. In order to implement this policy, Australia subsequently agreed to five national mental health plans, the latest of which was signed by all jurisdictions in 2017 [7]. A second National Policy was also produced [8].

Despite this apparent commitment to reform [6], it is worth noting that in 2017-18 there were still 1613 beds in psychiatric hospitals spread across five states, costing $565 millions or just under 10% of total state spending on mental health. Half of the remaining institutional beds are in NSW [9]. It is also notable that the current Fifth National Plan does not provide a definition of community mental health care and makes no reference to the term ‘hospital avoidance’. Recent changes to the way health services are funded have compounded confusion regarding the desired, ultimate goal of mental health reform. The application of tools such as Activity Based Funding has been seen by some to incentivize admitted care over other forms of care, including in relation to community mental health [10]. Others have even suggested that a core problem is in fact a lack of acute mental health hospital beds [11]. Across the country, the average length of stay in a mental health unit at a public hospital has been reducing, from 15.1 days in 2010-11 to 13.1 days in 2017-18 [9].

As a final contextual matter, it is important to understand that despite repeated policy concern and attention since 1992, expenditure on mental health has remained largely unchanged, from 7.3% of total health spending in 1992-93 compared with 7.6% in 2017-18 [12]. Data suggest that mental illness represents around 12% of the total burden of disease. While this gap between disease burden and expenditure may not entirely explain Australia’s systemic mental health problems, compared to other areas of health and given its contribution to Australians’ total burden of disease, mental health has clearly received relatively less funding. This makes the task of mental health reform more difficult.

## COMMUNITY MENTAL HEALTH IN AUSTRALIA

In the absence of a nationally agreed approach to community mental health, different perspectives or models have emerged. From the point of view of most states and territories, community mental health services typically comprise health professionals working in teams. These services, which might include psychiatrists, clinical and registered psychologists, mental health nurses and allied health professionals (such as occupational therapists and social workers), operate under a variety of names, such as community crisis teams, home care teams, (such as those based on the Assertive Community Treatment model), early psychosis intervention teams, youth mental health teams and residential rehabilitation units.

Effective community-based treatment typically entails the following: ready access to 24-hour crisis intervention and ongoing care, assertive and intensive community case management, professionally supervised residential treatment and rehabilitation in the community as an alternative to confining people to psychiatric institutions and real recovery-oriented vocational opportunities for individuals with mental illnesses [13]. There is evidence to suggest that community-centred health care of this nature is both more cost-efficient and cost-effective than hospital-centred care, particularly where community services are physically placed in the community and linked closely to both primary health care and hospital-based services [14].

In addition to this rather clinical definition, consumers (service users) and carers have also repeatedly expressed their views about a more holistic vision for the role community mental health care should play [15], including:

- actively managing medical and non-medical treatment for extended periods as required, with a focus on recovery;
- skilling people with mental illness to live independently in the community;
- providing access to and supporting accommodation and fulfilling employment opportunities, and other social and recreational activities;
- establishing and maintaining mental health centres or facilities that offer a range of support services and information;
- providing outreach services and home based assistance;
- providing case management that acknowledges the episodic nature of mental illness;
- providing timely access to graduated levels of assistance and intervention;
- services that respond quickly when someone is entering an episode of acute illness; and recognizing and offsetting the significant burden on families and carers through respite care.

### Variations in spending and trends

Spending on mental health is reported by the Australian Institute of Health and Welfare [12]. Drawing on these data, Table 1 shows state and territory spending on mental health care since 2007-08, by the percentage each key service component represents of total spending.

**Table 1:** Table 1. Variations in percentage spending between Australian states and territories across key mental health service components * NSW - New South Wales, VIC - Victoria, QLD - Qeensland, WA - Western Australia, SA - South Australia, TAS - Tasmania, ACT - Australian Capital Territory, NT - Nothern Territory

Years	NSW*	VIC	QLD	WA	SA	TAS	ACT	NT	Aust Average
Public psychiatric hospital									
2017-18	14.6	4.0	6.9	10.9	15.0	–	–	–	9.4
2011-12	17.0	4.0	10.9	15.3	18.6	-	-	-	11.9
2007-08	17.5	4.9	12.5	16.9	29.2	-	-	-	13.5
Public acute hospital									
2017-18	39.7	30.5	31.5	35.3	30.6	28.9	37.3	37.1	34.3
2011-12	36.5	27.1	30.0	28.8	21.5	37.7	24.5	32.9	30.7
2007-08	32.9	27.5	35.7	28.3	21.8	37.1	26.7	33.5	30.6
Total admitted patient									
2017-18	54.2	34.5	38.3	46.2	45.6	28.9	37.3	37.1	43.7
2011-12	53.5	31.1	40.9	44.0	40.1	37.7	24.5	32.9	42.5
2007-08	50.3	32.5	48.2	45.2	51.0	37.1	26.7	33.5	44.1
Community residential									
2017-18	0.5	14.1	4.0	3.7	7.3	25.5	10.5	10.0	6.2
2011-12	0.9	16.2	-	3.7	5.4	18.5	13.9	3.1	5.6
2007-08	1.5	16.3	-	2.3	2.3	21.0	12.4	1.3	5.7
Ambulatory									
2017-18	32.4	37.0	44.7	38.7	37.6	30.8	41.4	41.6	37.3
2011-12	35.7	38.9	45.0	41.3	42.2	31.9	44.7	47.9	39.7
2007-08	35.9	37.8	40.1	43.7	35.8	31.7	45.2	47.1	38.3
Non-government organizations									
2017-18	7.0	8.0	7.3	5.8	6.8	11.0	7.9	7.5	7.3
2011-12	5.0	8.3	7.8	5.5	9.8	6.1	13.3	7.3	6.9
2007-08	5.8	8.2	6.3	5.3	8.9	5.1	10.2	11.0	6.8
Indirect									
2017-18	5.8	6.3	5.6	5.5	2.7	3.8	2.9	3.9	5.5
2011-12	4.9	5.5	6.2	5.5	2.5	5.9	3.5	8.8	5.2
2007-08	6.4	5.2	5.3	3.4	2.1	5.2	5.5	7.0	5.2

Some trends are clear. The first is that spending on public acute services is an increasingly important element of spending nationally, now accounting for more than 35% of all spending. There are jurisdictional differences, which are further highlighted when considering public psychiatric hospitals as well as mental health services provided in general public hospitals. For example, in 2017-18, NSW spent 54% of total mental health expenditure on admitted care, while Victoria only spent 34%. The states also vary markedly in their approach to community residential spending. Key differences between jurisdictions in 2017-18 are circled in Table 1 for ease of reference.

Ambulatory services also vary between jurisdictions. However, analysis here is complicated by the fact that this label refers to a mix of services, including those provided in a range of hospital outpatient clinics, telephone calls, community visits and other matters. It is not possible to clearly divide those services listed as ‘ambulatory’ between those actually provided at hospital versus those genuinely available in the community or people’s homes.

While the percentage of total expenditure associated with ambulatory services has gone down over the past decade, the number of recorded services has grown appreciably from 5.66 millions in 2005-06 to 9.7 millions in 2018-19. However, the proportion of these ambulatory services taking less than 15 minutes per client has risen over this same period, from 38.6% to 44% and overall, the average duration of each recorded community mental health service has declined from 45 minutes to 35 minutes [16].

Interactions of this brevity suggest that an increasing proportion of so-called ambulatory care is in fact short, regular visits by patients to hospital outpatient clinics or telephone calls, rather than home visits or genuine community-based care. These data may reflect workforce capacity restrictions and growing demands on overstretched services, highlighted elsewhere [3]. They may also be consistent with recent trends in some jurisdictions, such as Victoria, to provide fewer home care and outreach services in the form of Assertive Community Treatment.

Table 1 also clearly demonstrates the peripheral nature of non-government organizations (NGOs) as part of the mental health service landscape. Unlike other places, for example, New Zealand, where spending on NGOs has been as high as 30% of total expenditure on mental health [17], in Australia this sector has languished at around 7%. This has deprived Australia of a range of psychosocial rehabilitation and support services, as alternatives to or as a means of minimizing prolonged or avoidable hospitalization. One explanation for this stunted growth is the early split between clinical and psychosocial support services, which arguably led to greater fragmentation of community-based services and less visibility for the important complementary role of these support services [18].

One practical manifestation of this split has been a reluctance to invest in a peer workforce in mental health. While these roles have become commonplace in other countries [19], in Australia in 2017-18, consumer workers in paid roles represented just 6.4 out of every 1,000 Full Time Equivalent employee in mental health, and carer workers 2.4 out of every 1,000 [9]. Australia’s response to mental illness continues to depend heavily on trained health professionals.

Again, unlike other countries [20], Australia maintains quite a strict and unhelpful delineation between clinical and non-clinical mental health services, with separate professional training arrangements. This makes holistic, comprehensive and multidisciplinary care less likely.

In addition to the state and territory resources described above, the federal government had begun to demonstrate greater interest in community mental health. Since 2006, it has made a large investment in public access to psychology services (now costing around $16 millions a week [21]) and in other programmes, like Partners in Recovery and Personal Helpers and Mentors, which aimed to improve access to and coordination of community-based services for Australians with mental health problems [22].

However, investment in community mental health by all Australian governments has now been affected by the implementation of the National Disability Insurance Scheme (NDIS). Akin to Australia’s investment in a national public health insurance scheme (Medicare), the country recently chose to address the lifelong costs associated with permanent and severe disability through a similar national insurance arrangement. Mental health was a late addition to the discussion about how to design the NDIS. Its eventual inclusion has not been straightforward.

Of most relevance to this analysis, however, was the decision by all nine governments to shift the funding associated with psychosocial mental health support services to the NDIS, as part of the initial set-up of the Scheme.

Australia’s psychosocial support service sector has always been a marginal element of the service landscape. Even in places like Victoria and the Australian Capital Territory (ACT), where the investment has been appreciably larger than in other jurisdictions, at their zenith these services only represented around 15% of total spending on mental health care. In NSW, it was more like 7%. However, the vast bulk of this spending has now been transferred to the NDIS and then to individualized care packages.

Community-managed organizations, some of which had been providing psychosocial community support services for decades, found that without the traditional block funding arrangements, they were not able to offer sustainable employment contracts to their staff.9 Ironically, while the NDIS has brought more and new funding to disability services, its impact in mental health care has been to lessen choice and availability of specialist psychosocial services, effectively excluding some people with manifest psychosocial disabilities.

### Key challenges for reform

Mental health remains a critical area of political and community concern, with widespread appreciation of systemic deficiencies. It is one of the most investigated areas of public policy in Australia; there were 32 separate statutory inquiries between 2006 and 2012 alone [23]. With three current Royal Commissions and one Productivity Commission inquiry underway or about to be completed, this trend continues.

A common finding of these past inquiries has been chronic underfunding of community-based mental health services. For example, the 2006 report by the Australian Senate suggested in response to this finding that Australia build around 200 community mental health centres [15].

While it is possible to point to some of these major trends affecting the development of community mental health services across the country, again it should be stressed that the picture varies between jurisdictions. At some periods, most jurisdictions have established some level of community mental health care. However, as shown in Table 1 and as reported recently by the Productivity Commission, efforts have generally been uneven, uncoordinated and unsustained [9]. Hospital-centred services continue to dominate. This has implications for the country’s mental health workforce and whether they have the training, skills, attitudes and motivation required to work in community settings [24].

While the Australian community and successive inquiries have identified the need for much greater investment in community mental health services, blending both clinical and psychosocial elements of care, the prevailing reality of 'community-based care' is limited, increasingly restricted to brief episodes and overly clinically-focussed compared with the needs and expectations of the community. There is evidence of a retreat from, or even dismantling of, community mental health services [14]. Too many services are being collocated with hospitals or provided out of hospitals, rather than in community settings. Opportunities for early intervention are lost.

Perhaps the first and most important thing Australia can do to arrest this costly and often traumatic situation is to re-assert the vision originally described in 1992, of a shared goal to enable people with mental illness to wherever possible, live with dignity in the community. Re-dedicating policy and funding efforts towards this shared goal would see home and community-based mental health care prioritized above hospital-based care. It would also see a better balance established between clinical and psychosocial needs [25], with the emphasis being on earlier intervention.

To this renewed vision should be added more practical pathway-type data, clearly demonstrating when and how community mental health care fits with primary and tertiary care. These data are not currently available and this lack of role clarity contributes to the vulnerability of community mental health services. The recent reallocation of responsibility for mental health planning to regional networks offers some new opportunities to develop this pathway [26].

However, reform must be supported by the right financial incentives, enabling community care to be prioritized over hospital-based mental health care and waiting times in Emergency Departments. Indeed, this would recognize that good community care can decrease re-admissions to hospital [27]. Regional reform must also seek to integrate funding from different sources, including the NDIS, in order to ensure that all components of community mental health care are available and can flourish.

Lastly, it would be prudent to ensure that this new prioritization of community mental health is supported by an effective and comprehensive process of accountability and governance [3]. Current systems are weak and do not permit a detailed understanding of the impact of care on the patient’s quality of life [28]. For the purpose of impelling systemic quality improvement in mental health, it is vital service providers can determine whether the care provided has resulted in effective outcomes and recovery.

More than 25 years after Australia’s first national mental health plan was produced, the establishment of a vibrant community mental health system remains the county’s greatest and most urgent challenge.

## Authors contribution

Dr. Rosenberg was the main author of the paper, with Professor Harvey providing input.

## Conflict of interest

the authors declare no conflict of interest.

## Funding

Neither author have received any funding associated with this article.

## References

[webpage-ref-581e8ac4c7ed5941aa006cc3ce00e9ed] (2017). Health System Performance Rankings.. The Commonwealth Fund.

[journal-article-ref-b051fe07a2640d854695c016219db42f] Rosenberg Sebastian, Hickie Ian (2018). No gold medals: Assessing Australia’s international mental health performance. Australasian Psychiatry.

[webpage-ref-66ae7d9ccd71d28c448528ab110ec5ac] (2019). Victorian Auditor General’s Office, Access to Mental Health Services, Independent Assurance Report to Parliament 2018–19: 16.. Victorian Government.

[journal-article-ref-42760c0e024bb5ef2a9c4d4d4cf132b5] Smullen Amanda (2015). Not Centralisation but Decentralised Integration through Australia's National Mental Health Policy. Australian Journal of Public Administration.

[book-ref-88c90060303446d9e31a0e90de5401d2] Meadows G, Farhall  J, Fossey  E (2012). Mental Health in Australia: Collaborative Community Practice.

[webpage-ref-938808b7aef52d9f0cf71841b3cf6715] Australian Health Ministers (1992). National mental health policy. Australian Health Ministers.

[webpage-ref-4bfbcdb24e5d9fd13151426f049c34cd] Council of Australian Governments (2019). The Fifth National Mental Health and Suicide Prevention Plan. 2017.

[webpage-ref-7394342d56844d0d831f7460ba00e215] Australian Government (2019). National mental health policy 2008.

[webpage-ref-708ba4d05af10728af12eb87b0a06063] (2020 ). Productivity Commission.. Report on Government Services 2020.

[webpage-ref-9534d1301578972cf021c179ae7fef54] NSW Mental Health Commission (2019). Review of transparency and accountability of mental health funding to health services. July 2017.

[journal-article-ref-9c77c408bf6368106f3f64d3e70a327e] Allison Stephen, Bastiampillai Tarun (2015). Mental health services reach the tipping point in Australian acute hospitals. Medical Journal of Australia.

[webpage-ref-bb5d93be6e682bd06212c3b787518888] Australian Institute of Health and Welfare (2020). Mental Health Services in Australia, Expenditure.

[journal-article-ref-17237ff09189413a28aaf8bf2cd7c1ab] Rosen A, Newton  L, Barfoot  K (2003). Evidence-based community alternatives to institutional psychiatric care. Medicine Today.

[journal-article-ref-b6498eaffefbb99746d2a9a042a30ef9] Rosen Alan, Gurr Roger, Fanning Paul (2010). The future of community-centred health services in Australia: lessons from the mental health sector. Australian Health Review.

[webpage-ref-7d6e95e6bd1185a30422ad762a409676] Parliament of Australia (2019). A national approach to mental health – from crisis to community. 2005.

[webpage-ref-bd72f5211898be5b52f857666f8c070d] Australian Institute of Health and Welfare (2020). Mental Health Services in Australia, Community Care Services.

[webpage-ref-81d9928fd9222e8a382bb4c7567e71e5] Platform Charitable Trust (2019). Frontline - The community mental health and addiction sector at work in New Zealand.

[journal-article-ref-acc180b6553db45e7cb4e5667c1efe43] Whiteford Harvey (1994). Intersectoral policy reform is critical to the National Mental Health Strategy. Australian Journal of Public Health.

[journal-article-ref-6082e41fd7742285d12b10d096c94091] Bennett Laura (2016). Support time recovery workers: 13 years on. Mental Health Practice.

[webpage-ref-b874770f07fb3053ed28a690a6a700c2] (2019). Te Pou o te Whakaaro Nu National centre of evidence based workforce development for the mental health, addiction and disability sectors in New Zealand.

[journal-article-ref-fd690497b8812c3a783df755c8782206] Rosenberg Sebastian P, Hickie Ian B (2019). The runaway giant: ten years of the Better Access program. Medical Journal of Australia.

[webpage-ref-111e37c9c27a1a0bf764a2e57d69e7d7] (2019). Australian Government. Psychosocial support for people with severe mental illness.

[book-ref-2b90df0c1d8dc331400a53f3be7f5acb] Mendoza  J, Bresnan  A, Rosenberg  S (2013). Obsessive Hope Disorder: Reflections on 30 Years of Mental Health Reform in Australia and Visions for the Future.

[webpage-ref-7ba8ac6748753e5f238c5753f4457a9f] Miller  M, Siggins  I, Ferguson  M (2019). National mental health workforce literature review. 2011. Melbourne, Department of Health.

[journal-article-ref-4bb0898b8587722a5f9fce6f7c0b958c] Harvey Carol, Brophy Lisa, Parsons Samuel, Moeller-Saxone Kristen, Grigg Margaret, Siskind Dan (2015). People living with psychosocial disability: Rehabilitation and recovery-informed service provision within the second Australian national survey of psychosis. Australian New Zealand Journal of Psychiatry.

[webpage-ref-f5e06bc54f28acfdbd7d86af45baa319] Capital Health Network (2019). Primary Health Networks (PHNs) and Mental Health Reform.

[journal-article-ref-dad4349e06e966d31feaa3cecff1deae] Zhang Jianyi, Harvey Carol, Andrew Carol (2011). Factors Associated with Length of Stay and the Risk of Readmission in an Acute Psychiatric Inpatient Facility: A Retrospective Study. Australian New Zealand Journal of Psychiatry.

[journal-article-ref-fa006f234aa4536443bcf6723469041a] Rosenberg Sebastian P, Hickie Ian B, McGorry Patrick D, Salvador‐Carulla Luis, Burns Jane, Christensen Helen, Mendoza John, Rosen Alan, Russell Lesley M, Sinclair Sally (2015). Using accountability for mental health to drive reform. Medical Journal of Australia.

